# Axillary management in patients with clinical node-negative early breast cancer and positive sentinel lymph node: a systematic review and meta-analysis

**DOI:** 10.3389/fonc.2023.1320867

**Published:** 2024-01-08

**Authors:** Changzai Li, Pan Zhang, Jie Lv, Wei Dong, Baoshan Hu, Jinji Zhang, Hongcheng Zhu

**Affiliations:** ^1^ Department of Oncological Surgery, North China University of Science and Technology Affiliated Hospital, Tangshan, Hebei, China; ^2^ College of Nursing and Rehabilitation, North China University of Science and Technology, Tangshan, Hebei, China

**Keywords:** breast cancer, sentinel lymph node biopsy, axillary lymph node dissection, axillary radiation, axillary management

## Abstract

**Background:**

The omission of axillary lymph node dissection (ALND) or axillary radiation (AxRT) remains controversial in patients with clinical node-negative early breast cancer and a positive sentinel lymph node.

**Methods:**

We conducted a comprehensive review by searching PubMed, Embase, Web of Science, and Cochrane databases (up to November 2023). Our primary outcomes were overall survival (OS), disease-free survival (DFS), locoregional recurrence (LRR), and axillary recurrence (AR).

**Results:**

We included 26 studies encompassing 145,548 women with clinical node-negative early breast cancer and positive sentinel lymph node. Pooled data revealed no significant differences between ALND and sentinel lymph node biopsy (SLNB) alone in terms of OS (hazard ratio [HR]0.99, 95% confidence interval [CI] 0.91-1.08, *p*=0.84), DFS (HR 1.04, 95% CI 0.90-1.19, *p*=0.61), LRR (HR 0.76, 95% CI 0.45-1.20, *p*=0.31), and AR (HR 1.01, 95% CI 0.99-1.03, *p*=0.35). Similarly, no significant differences were observed between AxRT and SLNB alone for OS (HR 0.57, 95% CI 0.32-1.02, *p*=0.06) and DFS (HR 0.52, 95% CI 0.26-1.05, *p*=0.07). When comparing AxRT and ALND, a trend towards higher OS was observed the AxRT group (HR 0.08, 95% CI 0.67-1.15), but the difference did not reach statistical significance (*p*=0.35, I^2^ = 0%). Additionally, no significant differences significance observed for DFS or AR (*p*=0.13 and *p*=0.73, respectively) between the AxRT and ALND groups.

**Conclusion:**

Our findings suggest that survival and recurrence rates are not inferior in patients with clinical node-negative early breast cancer and a positive sentinel lymph node who receive SLNB alone compared to those undergoing ALND or AxRT.

## Introduction

Axillary lymph node dissection (ALND) has been the standard therapeutic approach for breast cancer patients with positive sentinel lymph nodes. However, ALND is associated with various complications, including lymphedema, paresthesia, infections, axillary seromas, and other significant morbidities (1). Currently, sentinel lymph node biopsy (SLNB) is recommended for assessing axillary nodal lymph node status in early breast cancer patients who are clinically node-negative. The National Comprehensive Cancer Network (NCCN) suggests that ALND is not required for breast cancer patients with a negative sentinel node. The role of ALND for early-stage breast cancer patients with a limited number of metastatic sentinel lymph nodes remains controversial. According to the American Society of Clinical Oncology Clinical Practice Guideline, ALND should be considered for women with early breast cancer and one to two positive sentinel lymph nodes who are planning to undergo mastectomy (2). Pepels et al. indicated that ALND was recommended in patients with sentinel micrometastases and unfavorable tumor characteristics (3), while no ALND for patients with sentinel lymph nodes micrometastases resulted in a higher five -year regional recurrence rate compared to ALND (4).

However, Galimberti et al. suggest that ALND may be overtreatment for early-stage breast cancer patients, particularly when the tumor burden in the sentinel lymph nodes is minimal or moderate (5). NCCN also suggests that patients who have T1/T2 tumors, one to two positive sentinel lymph nodes, and plan to undergo whole-breast radiotherapy (RT) following breast-conserving therapy are not recommended for ALND (6). The ACOSOG Z0011 (American College of Surgeons Oncology Group) trial demonstrated that patients with clinical T1/T2 tumors and fewer than three positive sentinel lymph nodes undergoing lumpectomy and whole-breast radiation therapy could avoid ALND without negatively impacting local recurrence, disease-free survival, and overall survival (7).Additionally, the IBCSG 23-01 trial, designed to compare outcomes in patients with one or more sentinel micrometastases (≤2 mm) treated with ALND versus no ALND, showed no significant differences in five-year overall survival and five-year disease-free survival (8). A previous retrospective study also indicated that ALND did not improve either post-mastectomy overall survival or disease-free survival among breast cancer patients with one to three positive sentinel lymph nodes (9).

The AMAROS trial aimed to evaluate whether axillary radiation (AxRT) achieved better regional control and fewer side effects compared to ALND. Finding demonstrated that AxRT offered similar axillary control for patients with T1/T2 breast cancer and positive sentinel lymph nodes, while significantly reducing. the occurrence of lymphedema (10). A retrospective cohort study comparing patients with T1/T2 and less than two macrometastases (>2 mm) who underwent either AxRT or non-AxRT also observed similar overall and disease-free survival rates. There was no statistically significant difference in the five-year outcomes between the two groups (11). Motivated by these finding, we conducted a systematic review to compare outcomes in clinical node-negative early breast cancer patients with sentinel lymph node metastasis who underwent mastectomy or breast-conserving surgery. This meta-analysis aims to assess overall survival, disease-free survival, locoregional recurrence and axillary recurrence according to the type of axillary management(SLNB alone, ALND, or AxRT).

## Materials and methods

### Study selection

We conducted a systematic search of English literature in PubMed, Embase and Cochrane Library databases up to November 2023. Our search encompassed published data only. Search terms included keywords and MeSH terms such as “breast cancer/breast carcinoma”, “sentinel lymph node biopsy,” “axillary lymph node dissection”, and “axillary radiation,” Two authors (C.Z.L and P.Z) independently reviewed the available literature based on the inclusion criteria. Subsequently, potentially relevant references with sufficient information in their titles and abstracts were retrieved full-text article assessment. If the included studies were based on the same data, we selected the latest published version. Any disagreements were resolved through discussion and consensus among the authors. The study selection process adhered to the PRISMA guidelines.

### Study inclusion and exclusion criteria

The inclusion criterial were as follows: (1) Design: randomized controlled trials (RCTs) and retrospective studies. (2) Patient eligibility: Studies enrolling patients with clinical node-negative early breast cancer and positive sentinel lymph node (3) Comparative interventions: SLNB alone versus ALND, ALND versus AxRT, and SLNB alone versus AxRT. (4) Outcomes: Studies reporting on overall survival, disease-free survival, axillary recurrence, and locoregional recurrence. The exclusion criterial included abstracts, reviews, case reports, and articles deemed irrelevant or containing missing data.

### Data extraction and management

Following the Cochrane Handbook guidelines, two authors independently extracted data from the included studies. Recorded information included the authors’ names, publication year, number of participants, study design, intervention type, tumor stage, micometastasis or macrometastsis count, adjuvant radiation therapy, follow-up duration (years), outcomes, and the quality of evidence in each study. Any discrepancies were addressed and resolved through discussion or with the assistance of a third author.

### Quality assessment in individual studies

The risk of bias in all included studies was evaluated using guidelines in the Cochrane Handbook for Systematic Reviews of Interventions (https://training.cochrane.org/handbooks) (12). Two authors independently assessed the potential risk of bias, including selection bias, performance bias, detection bias, attrition bias, reporting bias, and confounding bias, and other sources of bias. For RCTs, the GRADEpro GDT (Grading of Recommendation Assessment Development and Evaluation Profiler Guide line Development Tool)was used to assess evidence quality. This online too, available at https://www.gradepro.org, evaluates five factors: risk of bias, inconsistency, indirectness, imprecision, and other considerations. Based on these factors, evidence quality is classified into four levels: high (⊕⊕⊕⊕), moderate (⊕⊕⊕⊖), low (⊕⊕⊖⊖) or very low (⊕⊖⊖⊖). For non-RCTs, the Newcastle-Ottawa Scale (NOS) served as the quality assessment tool (13). The NOS awards stars based on three domains: quality of patient selection (up to four stars), comparability between cases and controls (up to two stars), and adequate ascertainment of exposure (up to three stars). Studies with more than seven stars were considered to have a high level of evidence. Two authors independently assessed the quality of evidence in the included studies, With any disagreements resolved through discussion.

### Statistical analysis

We used the Review Manager software (version 5.4), update by the Cochrane Library for Systematic Review, to perform the analysis. The summary statistic of generic inverse variance (overall survival, disease-free survival, axillary recurrence, and locoregional recurrence) was assessed by hazard ratios (HRs). 95% confidence intervals (CIs) were calculated using the fixed-effect model. The statistical heterogeneity of the included studies was quantified and examined using the I^2^ statistics. An I^2^ value of 0% to 25% indicates low heterogeneity, 25% to50% indicates moderate heterogeneity, 50% to75% indicates large heterogeneity, and 75% to100% indicates huge heterogeneity (14). When heterogeneity was observed, we employed the random-effects model. We conducted the subgroup analysis based on the type of axillary management (SLNB alone, ALND, and AxRT). Sensitivity analysis was performed to identify sources of heterogeneity. A funnel plot was used to assess publication bias in the included studies. A p value less than 0.05 was considered statistically significant.

## Results

### Study selection

**Figure 1:**
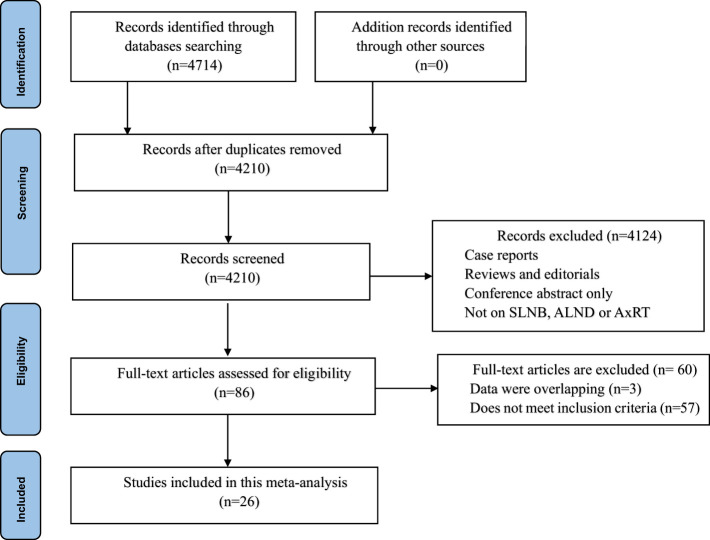
Flow diagram of study selection.

Our initial database search yielded a total of 4,714 studies. After removing 504 duplicate studies, we screened the titles and abstracts of 4,210remaining studies. A total of 4,124 studies were excluded due to irrelevance (non-related studies, review articles, case reports, meta-analysis, or lack of data). At the full-text level, 86 potentially eligible studies were assessed, t of which 60 were ultimately excluded after a thorough review. This left 26 studies involving 145,548 patients for inclusion in the meta-analysis (5, 7, 11, 15–36) The PRISMA flowchart in **Figure 1** illustrates this selection process.

### Study characteristics

**Table 1** outlines the characteristics of the included studies. Eighteen studies were retrospective cohort studies (11, 18–27, 34–36), while the remaining eight were RCTs (5, 7, 28–33). The studies were published between April 2009 to July 2023.Sample sizes range from 121 to 97,314 patients, with a total of 145,548 patients analyzed. Among these patients, 40,156 received SLNB alone, while 105,418 underwent either ALND or AxRT. All included studies were published in English. Twenty-two studies reported overall survival data (5, 7, 11, 15–27, 29, 30, 32, 33, 35). Eleven studies reported disease-free survival data (5, 7, 11, 15, 23, 30–35). Six studies analyzed locoregional recurrence (21, 26, 28, 35, 36), and eight studies reported axillary recurrence (11, 22, 24, 26, 30, 32, 33, 35).

**Table 1:** Summary of characteristics of included studies.

Reference	Type of study	SLNB alone/ALND or AxRT	T stage (T1/T2)	Micro/Macro	Adjuvant RT (Yes/No)	Follow up (years)	Outcomes	Quality (NOS^a^)
			SLNB alone ALND	SLNB alone ALND	SLNB alone ALND			
Tinterri 2023 (29)	RCT	107/218	53/51 47/56	NA NA	NA NA	2.8	OS	RCT
Houvenaeghel 2023 (23)	Retrospective	185/1266	NA NA	NA NA	1123/32 174/9	5.8	OS,DFS	6
Campbell 2023 (32)	RCT	544/544	NA NA	NA NA	482/62 466/73	10	OS,DFS,AR	RCT
Bartels 2023 (30)	RCT	681/744	533/143 612/132	195/419 215/442	681/0 703/41	10	OS,DFS,AR	RCT
Zhou 2022 (19)	Retrospective	1883/1883	740/878 725/862	1883/0 1883/0	596/1287 621/1262	4	OS	7
Kantor 2022 (34)	Retrospective	79/42	48/31 22/20	23/56 9/33	61/18 27/15	2.0	DFS, LRR	6
Sun 2021 (21)	Retrospective	128/201	62/58 82/101	NA NA	68/60 108/93	4.2	OS,LRR	7
Lim 2021 (35)	Retrospective	92/168	41/51 70/28	92/0 168/0	31/61 46/122	5.1	OS, DFS, LRR, AR	7
Ortega 2021 (11)	Retrospective	167/93	NA NA	0/167 0/93	95/72 NA/NA	4.5	OS, DFS, AR	6
Kim 2020 (16)	Retrospective	179/704	83/96 326/378	NA NA	NA NA	4.5	OS	8
Arisio 2019 (26)	Retrospective	211/406	118/82 211/189	155/95 84/322	169/42 335/71	7.0	OS, AR	6
Lee 2018 (20)	Retrospective	1268/3174	NA NA	NA NA	NA NA	3.9	OS	6
Galimberti 2018 (5)	RCT	469/465	322/140 316/142	NA NA	NA NA	9.7	OS, DFS, LRR	RCT
Wu 2018 (37)	Retrospective	11368/2651	4617/6751 1444/1207	NA NA	NA NA	1.9	OS	4
Savolt 2017 (33)	RCT	230/244	157/73 152/92	NA NA	230/0 0/244	8.1	OS, DFS, AR	RCT
Mamtani 2017 (36)	Retrospective	162/190	NA NA	NA NA	NA NA	6.0	LRR	6
Giuliano 2017 (7)	RCT	436/420	303/126 284/134	NA NA	NA NA	9.3	OS, DFS	RCT
Giuliano 2016 (28)	RCT	436/420	303/126 284/134	NA NA	NA NA	9.3	LRR	RCT
Tvedskov 2015 (24)	Retrospective	240/1834	NA NA	NA NA	NA NA	6.3	OS, AR	5
Snow 2015 (17)	Retrospective	60/258	NA NA	NA NA	36/24 147/111	6.3	OS	5
Bonneau 2015 (18)	Retrospective	402/9119	174/228 3665/5454	NA NA	192/210 5426/3677	2.6	OS	4
Park 2014 (27)	Retrospective	197/2384	130/67 1171/1177	NA NA	4/55 439/757	3.5	OS	5
Fu 2014 (25)	Retrospective	106/108	49/47/7 25/53/26	NA NA	59/46 65/28	3.6	OS	4
Yi 2013 (15)	Retrospective	188/673	152/36 445/228	136/52 158/515	NA NA	5.8	OS, DFS	7
Solá 2013 (31)	RCT	121/112	NA NA	NA NA	NA NA	5.0	DFS	RCT
Bilimoria 2009 (22)	Retrospective	20217/77097	NA NA	3674/16543 6585/70512	NA NA	7.9	OS,AR	6

RCT, randomized controlled trial; SLNB, sentinel lymph nodes biopsy; ALND, axillary lymph node dissection; OS, overall survival; LLR, locoregional recurrence; DFS, disease-free survival; AR, axillary recurrence; NOS, Newcastlee-Ottawa Scale; NA, not available; Micro, micrometasis (<0.2-2.0 mm); Macro, macromeataiss (>2.0 mm). RT, radiation therapy.

a Quality assessment of the observational studies was assessed using the Newcastlee-Ottawa Scale. The quality of the evidence is classified as three levels: high (more than seven stars), moderate (four to six stars), poor (less than four stars).

### Quality assessment

The NOS was used to assess the quality of evidence in non-RCT trials. Five studies received a rating of seven or more stars, indicating high quality (15, 16, 19, 21, 35).Seven studies received a rating of six stars (11, 20, 22, 23, 26, 34, 36), and six studies were assessed as having four to five stars (See **Table 1**). The GRADEpro GDT tool was used to classify the evidence of RCTs comparing ALND to SLNB alone for patients with clinical node-negative early-stage breast cancer and positive sentinel lymph nodes. The quality of evidence was high for disease-free survival and locoregional recurrence, and moderate in overall survival (See **Table 2**).

**Table 2:** Evaluating the quality of evidence in randomized controlled trials by GRADEpro GDT ALND compared to SLNB alone for patients with clinical node-negative early breast cancer and positive sentinel lymph node.

Patient or population: patients with clinical node-negative early breast cancer and positive sentinel lymph node Setting: Hospital Intervention: ALND Comparison: SLNB alone						
Outcomes	Anticipated absolute effects^*^ (95% CI)		Relative effect (95% CI)	№ of participants (studies)	Certainty of the evidence (GRADE)	Comments
	Risk with SLNB	Risk with ALND				
OS – Randomized control trials	514 per 1,000	**545 per 1,000** (485 to 603)	**HR 1.09** (0.92 to 1.28)	6406 (4 RCTs)	⊕⊕⊕◯ Moderate^a^	
DFS - Randomized control trials	512 per 1,000	**522 per 1,000** (472 to 574)	**HR 1.03** (0.89 to 1.19)	6872 (5 RCTs)	⊕⊕⊕⊕ High	
LRR - Randomized control trials	494 per 1,000	**400 per 1,000** (239 to 615)	**HR 0.75** (0.40 to 1.40)	3580 (2 RCTs)	⊕⊕⊕⊕ High	
*The risk in the intervention group (and its 95% confidence interval) is based on the assumed risk in the comparison group and the **relative effect** of the intervention (and its 95% CI). **ALND,** axillary lymph node dissection**; SLNB,** sentinel lymph node biopsy; **CI,** confidence interval; **HR,** hazard Ratio; **GRADEpro GDT**: Grading of Recommendation Assessment Development and Evaluation Pprofiler Guide- line Development Tool.						
**GRADE Working Group grades of evidence** **High certainty:** we are very confident that the true effect lies close to that of the estimate of the effect. **Moderate certainty:** we are moderately confident in the effect estimate: the true effect is likely to be close to the estimate of the effect, but there is a possibility that it is substantially different. **Low certainty:** our confidence in the effect estimate is limited: the true effect may be substantially different from the estimate of the effect. **Very low certainty:** we have very little confidence in the effect estimate: the true effect is likely to be substantially different from the estimate of effect.						

a.I^2^ value is 42% as moderate heterogeneity.

ALND, axillary lymph node dissection; SLNB, sentinel lymph nodes biopsy; OS, overall survival; LLR, locoregional recurrence; DFS, disease-free survival; CI, confidence interval; HR, hazard Ratio. The evidence quality was classified into 4 levels: high (⊕⊕⊕⊕), moderate (⊕⊕⊕⊖), low (⊕⊕⊖⊖) or very low (⊕⊖⊖⊖).

### Effect of ALND versus SLNB alone

**Figure 2:**
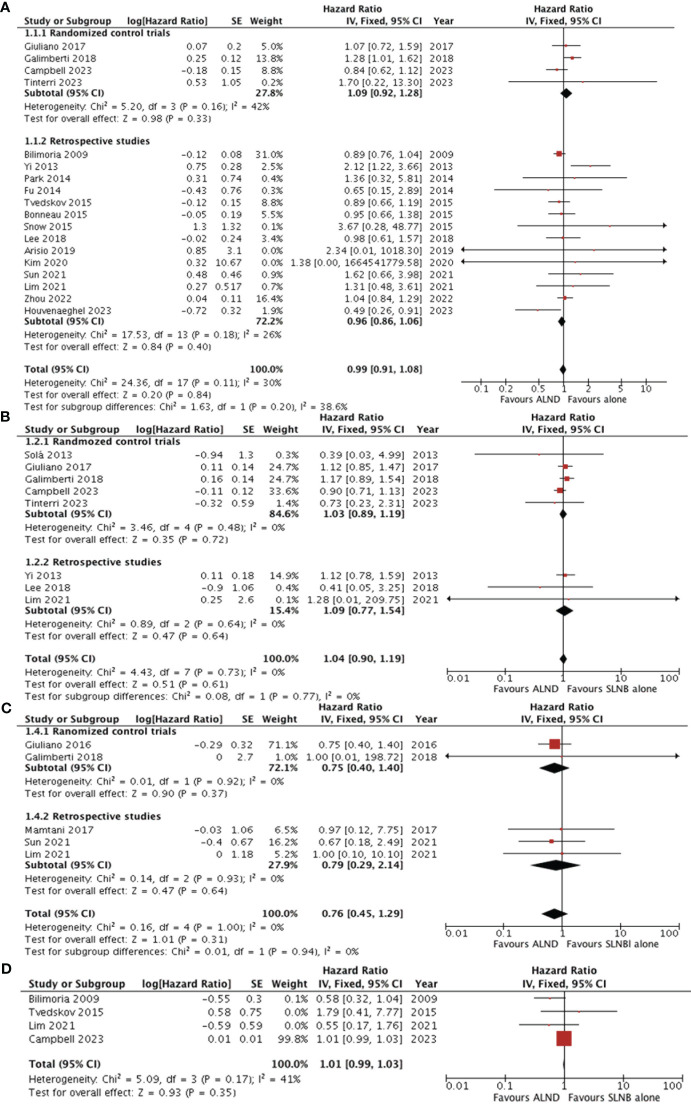
Forest plot of ALND versus SLNB alone **(A)** overall survival **(B)** disease-free survival **(C)** locoregional recurrence **(D)** axillary recurrence. ALND, axillary lymph node dissection; SLNB, sentinel lymph node biopsy; CI, confidence interval; SE, standard error; IV, Inverse Variance.

**Figure 3:**
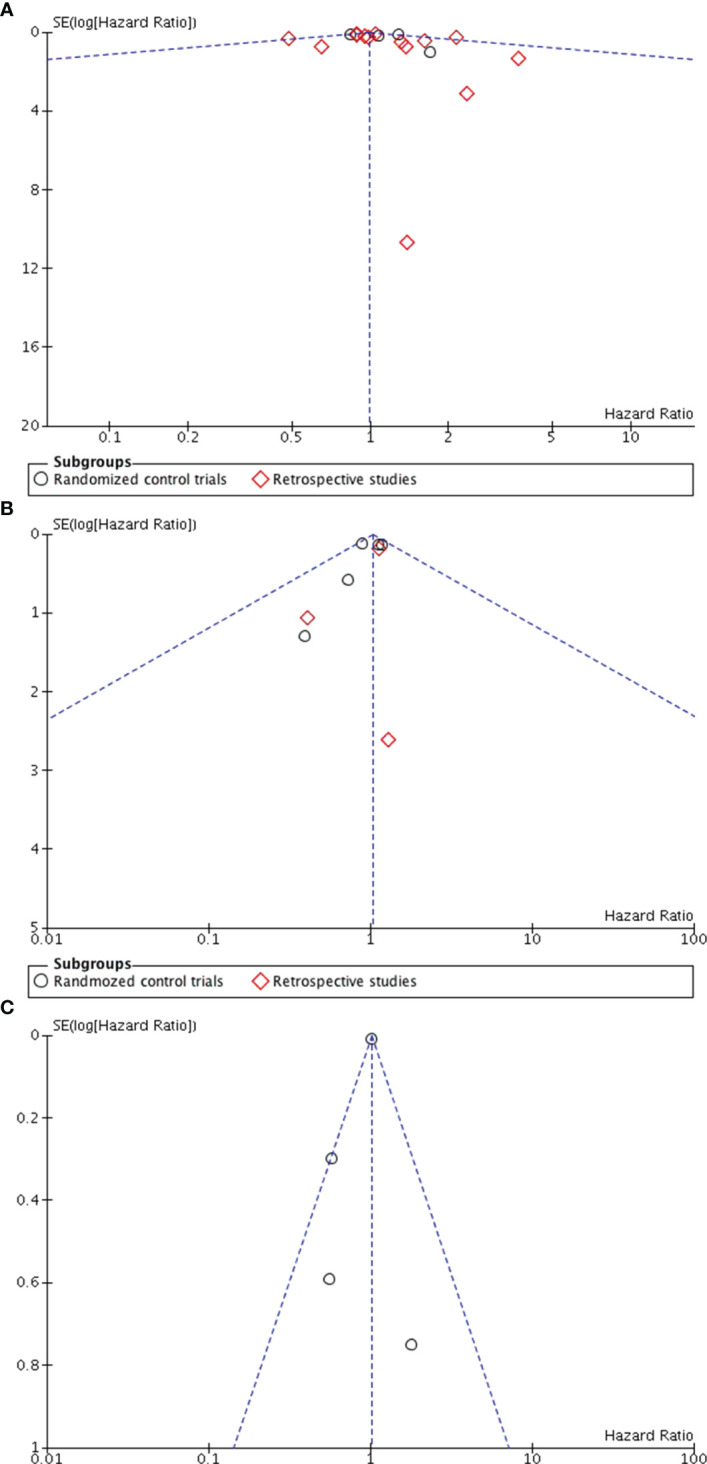
Funnel plot in ALND versus SLNB alone **(A)** Funnel plot for overall survival **(B)** Funnel plot for disease-free survival **(C)** Funnel plot for locoregional recurrence.

Eighteen studies (5, 7, 15–27, 29, 32, 35) reported the overall survival data for patients with SLNB alone versus ALND. The pooled results revealed no statistically significant difference between the groups (HR0.99, 95% CI0.91-1.08; *p*=0.84), and no significant heterogeneity was observed (I^2^ = 30%, p=0.11) (**Figure 2A**). A potential publication bias was suggested by the the asymmetry of the funnel plot (**Figure 3A**), Subgroup analysis showed no statistically significant difference in overall survival between SLNB alone and ALND in either RCTs (HR 1.09, 95% CI:0.92-1.28; p=0.33) or retrospective studies (HR 0.96, 95% CI:0.86-1.06; *p*=0.40). Data pooled from the eight studies (5, 7, 15, 20, 29, 31, 32, 35) demonstrated no substantial difference in disease-free survival between the SLNB alone group and ALND groups (HR 1.04, 95%CI:0.90-1.19; p=0.61). Additionally, the studies showed no significant heterogeneity (I^2^ = 0%, *p*=0.73) (**Figure 2B**). The funnel plot suggested the presence of publication bias (**Figure 3B**). Subgroup analysis revealed no significant difference in disease-free survival between RCTs (HR 1.03, 95%CI:0.89-1.19; *p*=0.72) or retrospective studies (HR 1.09, 95%CI:0.77-1.54; *p*=0.64).

Five studies evaluated locoregional recurrence (5, 21, 28, 35, 36). Pooled data indicated no statistically significant difference between patients who received SLNB alone and those who underwent ALND (HR 0.76, 95%CI 0.45-1.29; *p* = 0.31) (**Figure 2C**).However, the asymmetry of the funnel plot (**Figure 3C**) suggests potential publication bias.

Four studies reported the five-year cumulative incidence of axillary recurrence (22, 24, 32, 35). Although the axillary recurrence rate was higher in the SLNB alone group compared to the ALND group, but this difference was not statistically significant (HR1.01, 95% CI 0.99-1.03; *p* = 0.35) (**Figure 2D**).Additionally, the studies showed no significant heterogeneity (I^2^ = 41%, *p* = 0.17). However, only one study reported the 10-year axillary recurrence outcome (32). This study found that axillary recurrence was more frequent among those who received SLNB alone compared to ALND (HR 5.47, 95% CI 1.21-24.63; *p*=0.013).

### Effect of ALND versus AxRT

**Figure 4:**
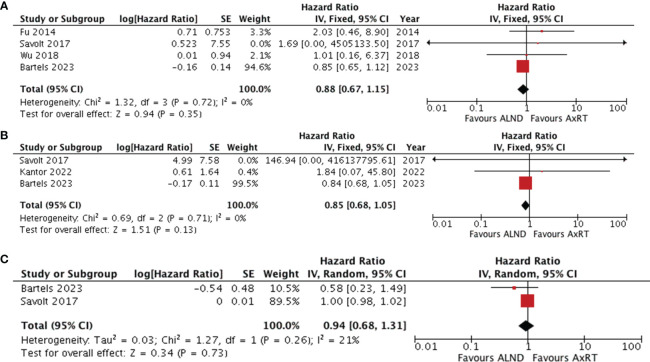
Forest plot of ALND versus AxRT **(A)** overall survival **(B)** disease-free survival **(C)** axillary recurrence. ALND, axillary lymph node dissection; AxRT, axillary radiation; CI, confidence interval; SE, standard error; IV, Inverse Variance.

Four studies (25, 30, 33, 37) compared overall survival between ALND and AxRT. Pooled data analysis revealed no significant difference between the two groups (HR 0.88, 95% CI: 0.67-1.15; *p*=0.35) (**Figure 4A**). Additionally, pooling data from three studies (30, 33, 34) assessing disease-free survival also showed no significant difference between ALND and AxRT (HR 0.85, 95% CI: 0.68-1.05; *p*=0.13). Furthermore, these studies exhibited no significant heterogeneity(I^2^ = 0%, p=0.71) (**Figure 4B**). Two studies (30, 33) reported axillary recurrence. While the axillary recurrence rate was higher in the AxRT group compared to the ALND group, the difference was not statistically significant (HR 0.94, 95% CI: 0.68-1.31; *p*=0.73) (**Figure 4C**). There was also no significant between-study heterogeneity(I^2^ = 21%, *p*=0.26).

### Effect of AxRT versus SLNB alone

**Figure 5:**
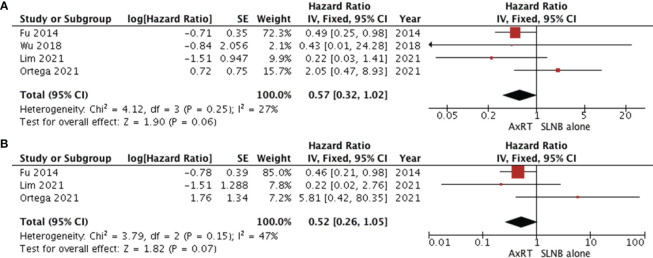
Forest plot of AxRT versus SLNB lone **(A)** overall survival **(B)** disease-free survival. AxRT, axillary radiation; SLNB, sentinel lymph node biopsy; CI, confidence interval; SE, standard error; IV,Inverse Variance.

Four studies (11, 25, 35, 37) assessed overall survival by comparing the AxRT group to the SLNB alone group. The results revealed no statistical difference in overall survival between the groups (HR 0.57, 95% CI: 0.32-1.02; *p*=0.25), with moderate heterogeneity between studies (χ2 = 4.12, I^2^ = 27%, *p*=0.27) (**Figure 5A**). The asymmetry of the funnel plot suggests publication bias.

Three studies (11, 25, 35) reported disease-free survival. Patients who received AxRT had a higher rate of disease-free survival than those who underwent SLNB alone (HR 0.52, 95% CI: 0.26-1.05).However, no statistically significant difference was observed between groups (*p*=0.07). There was moderate heterogeneity among studies (χ2 = 3.79, I^2^ = 47%, *p*=0.15) as shown in **Figure 5B**.

## Discussion

This meta-analysis aimed to compare the effects of SLNB alone, ALND, and AxRT on various outcomes, including overall survival, disease-free survival, locoregional recurrence, and axillary recurrence, in 145,548 patients with early-stage breast cancer, clinical negative axillary lymph nodes, and positive sentinel lymph nodes. The collected data revealed no significant differences in overall survival, disease-free survival, locoregional recurrence, and axillary recurrence between the SLNB alone group and the ALND or AxRT groups. While the AxRT group showed a higher overall survival rate compared to the ALND group, this difference was not statistically significant. Additionally, no significant disparities were observed in terms of overall survival and disease-free survival between patients who received AxRT and those who received SLNB alone.

Several meta-analyses (38–47) have been conducted to compare the differences in overall survival, disease-free survival, and recurrence rates between ALND and SLNB alone in early-stage breast cancer patients with positive sentinel lymph nodes. However, the impact of ALND remains controversial. Peristeri et al. (38) performed a meta-analysis comparing the effects of SLNB/RT and ALND in five RCTs. Their pooled data showed that the SLNB/RT group had better overall and disease-free survival than the ALND group, with a statistically significant difference in axillary recurrence favoring the ALND group. However, our previous meta-analysis comparing the two approaches in early-stage breast cancer with sentinel lymph node metastasis (43), found no significant differences in overall survival, disease-free survival and locoregional recurrence between the SLNB alone and the ALND group. Similarly, a meta-analysis of Real-World Evidence in the Post-ACOSOG Z0011 trial (39), which included one RCT and six retrospective studies with 8,864 early-stage breast cancer patients with one or two SLN metastases, found no differences between SLNB alone and ALND groups in overall survival, disease-free survival, and recurrence rate. However, the incidence rate of lymphedema was significantly lower in SLNB alone group Our systematic review and meta-analysis included eight RCTs and eighteen retrospective cohort studies. The results indicated no statistically significant difference in disease-free survival, overall survival, and locoregional recurrence between ALND and SLNB alone in clinical node-negative early breast cancer patients with positive sentinel lymph nodes. Three studies reported the five-year cumulative incidence of axillary recurrence. The rate was higher in the SLNB alone group compared to the ALND group, but this difference was not statistically significant (*p* = 0.36). However, when the follow-up period was extended to 10 years, Campbell et al. (32) found that that axillary recurrence was more frequent in the SLNB alone group compared to ALND group. Therefore, longer follow-up times are required to definitively compare the axillary recurrence rates between the two groups in this patients population. After ten year of follow-up, the Randomized Controlled EORTC AMAROS trials (30) showed that both AxRT and ALND groups achieved excellent locoregional control and survival in cT1-T2 breast cancer patients with positive sentinel lymph nodes. Additionally, the AxRT group had a lower rate of lymphedema and no difference in quality of life compared to the ALND group. This meta-analysis also confirms no significant differences in disease-free survival, overall survival, and axillary recurrence between patients treated with ALND versus AxRT.

Our review diverges from previous studies in several key aspects. First, by incorporating eight RCTs and 18 retrospective studies involving 145,548 patients, our meta-analysis significantly increase the sample size, leading to more precise and reliable results. Second, we employed the GRADEpro GTD tool to assess evidence quality within RCTs, revealing high quality for disease-free survival and locoregional recurrence, and moderate quality for overall survival. Furthermore, subgroup analyses based on study type (RCT vs. retrospective) demonstrated that the pooled analysis results remained unchanged, indicating the stability of our finding. Third, and uniquely, our review evaluated the effects of AxRT and SLNB alone in patients with clinical node-negative early breast cancer and positive sentinel lymph nodes, an aspect lacking in prior meta-analysis. However, limitations exist within our meta-analysis. First, our inclusion criteria restricted us to English studies only, potentially introducing publication bias by excluding unpublished data. Second, the NOS tool revealed six non-RCT studies with a, four to five-star rating, including lower-quality evidence. Third, both overall and disease-free survival analyses showed evidence of publication bias. Fourth, moderate heterogeneity was observed among studies regarding disease-free and overall survival when comparing the AxRT and SLNB alone groups. A study by Ortega et al. (11) identified potential sources of this heterogeneity. While removing their study resulted in a significant decrease in heterogeneity for both outcomes, the overall and disease-free survival data also changed substantially(**Supplementary Figures 1**, **2**). Therefore, further careful evaluation of the effect of AxRT versus SLNB alone is required, and these results necessitate confirmation through well-designed prospective studies. Finally, because the lack of sufficient information within the included studies precluded further subgroup analyses based on factors like the T1/T2 stage, number of positive sentinel lymph nodes, micrometastasis or macrometastasis, molecular subtype, and age.

## Conclusion

This study demonstrates that patients with early-stage breast cancer and positive sentinel lymph nodes who undergo SLNB alone achieve comparable locoregional control and survival to those who receive ALND or AxRT. Our findings suggest that omitting ALND or AxRT may be safe for these patients, although further verification is needed through rigorously designed prospective studies.

## Data availability statement

The original contributions presented in the study are included in the article/**Supplementary Material**. Further inquiries can be directed to the corresponding author.

## Author contributions

PZ: Data curation, Formal Analysis, Methodology, Writing – review & editing. CL: Data curation, Investigation, Methodology, Writing – original draft, Writing – review & editing. JL: Data curation, Investigation, Project administration, Writing – review & editing. WD: Formal Analysis, Methodology, Writing – review & editing. BH: Investigation, Methodology, Project administration, Software, Supervision, Writing – review & editing. JZ: Data curation, Investigation, Methodology, Writing – review & editing. HZ: Investigation, Methodology, Project administration, Software, Supervision, Writing – review & editing.

## Supplementary material

Supplementary Figure 1 Forest plot of overall survival after heterogeneity analysis in AxRT versus SLNB lone.

Supplementary Figure 2 Forest plot of disease-free survival after heterogeneity analysis in AxRT versus SLNB lone.
